# Supplementary material for: HIVprotI: an integrated web based platform for prediction and design of HIV proteins inhibitors
Source: J Cheminform. 2018 Mar 9;10:12. doi: 10.1186/s13321-018-0266-y (PMC5845081; doi:10.1186/s13321-018-0266-y)
Supplement: Supplementary file 2 — Additional file 2. Source code of HIVProtI web server. [file 13321_2018_266_MOESM2_ESM.zip › HIVprotI_Source-code/hivprotI/weka-3-6-6/doc/weka/datagenerators/DataGenerator.html]

DataGenerator


---


|  |  |  |
| --- | --- | --- |
| **Overview** \| **Package** \| **Class** \| **Tree** \| **Deprecated** \| **Index** \| **Help** \| **Weka's home** | |  |
| **PREV CLASS**   **NEXT CLASS** | **FRAMES**    **NO FRAMES**     **All Classes** |  |
| SUMMARY: NESTED \| FIELD \| CONSTR \| METHOD | DETAIL: FIELD \| CONSTR \| METHOD |  |


---


## weka.datagenerators Class DataGenerator

```
java.lang.Object
  weka.datagenerators.DataGenerator
```

**All Implemented Interfaces:**: java.io.Serializable, OptionHandler, Randomizable, RevisionHandler

**Direct Known Subclasses:**: ClassificationGenerator, ClusterGenerator, RegressionGenerator

---

``` public abstract class DataGenerator extends java.lang.Object implements OptionHandler, Randomizable, java.io.Serializable, RevisionHandler ```

Abstract superclass for data generators that generate data for
classifiers and clusterers.

**Version:**
:   $Revision: 1.8 $

**Author:**
:   FracPete (fracpete at waikato dot ac dot nz)

**See Also:**: Serialized Form

---

**Constructor Summary**

`DataGenerator()`
  
          initializes with default settings.


| **Method Summary** | |
| --- | --- |
| `java.lang.String` | `debugTipText()`             Returns the tip text for this property |
| `java.io.StringWriter` | `defaultOutput()`             Gets the string writer, which is used for outputting to stdout. |
| `Instances` | `defineDataFormat()`             Initializes the format for the dataset produced. |
| `java.lang.String` | `formatTipText()`             Returns the tip text for this property |
| `abstract  Instance` | `generateExample()`             Generates one example of the dataset. |
| `abstract  Instances` | `generateExamples()`             Generates all examples of the dataset. |
| `abstract  java.lang.String` | `generateFinished()`             Generates a comment string that documentates the data generator. |
| `abstract  java.lang.String` | `generateStart()`             Generates a comment string that documentates the data generator. |
| `Instances` | `getDatasetFormat()`             Gets the format of the dataset that is to be generated. |
| `boolean` | `getDebug()`             Gets the debug flag. |
| `int` | `getNumExamplesAct()`             Gets the number of examples the dataset should have. |
| `java.lang.String[]` | `getOptions()`             Gets the current settings of the datagenerator RDG1. |
| `java.io.PrintWriter` | `getOutput()`             Gets the print writer. |
| `java.util.Random` | `getRandom()`             Gets the random generator. |
| `java.lang.String` | `getRelationName()`             Gets the relation name the dataset should have. |
| `int` | `getSeed()`             Gets the random number seed. |
| `abstract  boolean` | `getSingleModeFlag()`             Return if single mode is set for the given data generator mode depends on option setting and or generator type. |
| `java.util.Enumeration` | `listOptions()`             Returns an enumeration describing the available options. |
| `static void` | `makeData(DataGenerator generator, java.lang.String[] options)`             Calls the data generator. |
| `java.lang.String` | `outputTipText()`             Returns the tip text for this property |
| `java.lang.String` | `randomTipText()`             Returns the tip text for this property |
| `java.lang.String` | `relationNameTipText()`             Returns the tip text for this property |
| `java.lang.String` | `seedTipText()`             Returns the tip text for this property |
| `void` | `setDatasetFormat(Instances newFormat)`             Sets the format of the dataset that is to be generated. |
| `void` | `setDebug(boolean debug)`             Sets the debug flag. |
| `void` | `setOptions(java.lang.String[] options)`             Parses a list of options for this object. |
| `void` | `setOutput(java.io.PrintWriter newOutput)`             Sets the print writer. |
| `void` | `setRandom(java.util.Random newRandom)`             Sets the random generator. |
| `void` | `setRelationName(java.lang.String relationName)`             Sets the relation name the dataset should have. |
| `void` | `setSeed(int newSeed)`             Sets the random number seed. |

**Methods inherited from class java.lang.Object**

`equals, getClass, hashCode, notify, notifyAll, toString, wait, wait, wait`

**Methods inherited from interface weka.core.RevisionHandler**

`getRevision`

**Constructor Detail**

### DataGenerator

```
public DataGenerator()
```

:   initializes with default settings.


**Method Detail**

### listOptions

```
public java.util.Enumeration listOptions()
```

:   Returns an enumeration describing the available options.

    :   **Specified by:**: `listOptions` in interface `OptionHandler`
    :   **Returns:**: an enumeration of all the available options

---


### setOptions

```
public void setOptions(java.lang.String[] options)
                throws java.lang.Exception
```

:   Parses a list of options for this object.

    For list of valid options see class description.

    :   **Specified by:**: `setOptions` in interface `OptionHandler`
    :   **Parameters:**: `options` - the list of options as an array of strings **Throws:**: `java.lang.Exception` - if an option is not supported

---


### getOptions

```
public java.lang.String[] getOptions()
```

:   Gets the current settings of the datagenerator RDG1. Removing of
    blacklisted options has to be done in the derived class, that defines
    the blacklist-entry.

    :   **Specified by:**: `getOptions` in interface `OptionHandler`
    :   **Returns:**: an array of strings suitable for passing to setOptions **See Also:**: `removeBlacklist(String[])`

---


### defineDataFormat

```
public Instances defineDataFormat()
                           throws java.lang.Exception
```

:   Initializes the format for the dataset produced.
    Must be called before the generateExample or generateExamples
    methods are used. Also sets a default relation name in case
    the current relation name is empty.

    :   **Returns:**: the format for the dataset **Throws:**: `java.lang.Exception` - if the generating of the format failed **See Also:**: `defaultRelationName()`

---


### generateExample

```
public abstract Instance generateExample()
                                  throws java.lang.Exception
```

:   Generates one example of the dataset.

    :   **Returns:**: the generated example **Throws:**: `java.lang.Exception` - if the format of the dataset is not yet defined: `java.lang.Exception` - if the generator only works with generateExamples which means in non single mode

---


### generateExamples

```
public abstract Instances generateExamples()
                                    throws java.lang.Exception
```

:   Generates all examples of the dataset.

    :   **Returns:**: the generated dataset **Throws:**: `java.lang.Exception` - if the format of the dataset is not yet defined: `java.lang.Exception` - if the generator only works with generateExample, which means in single mode

---


### generateStart

```
public abstract java.lang.String generateStart()
                                        throws java.lang.Exception
```

:   Generates a comment string that documentates the data generator.
    By default this string is added at the beginning of the produced output
    as ARFF file type, next after the options.

    :   **Returns:**: string contains info about the generated rules **Throws:**: `java.lang.Exception` - if the generating of the documentation fails

---


### generateFinished

```
public abstract java.lang.String generateFinished()
                                           throws java.lang.Exception
```

:   Generates a comment string that documentates the data generator.
    By default this string is added at the end of the produced output
    as ARFF file type.

    :   **Returns:**: string contains info about the generated rules **Throws:**: `java.lang.Exception` - if the generating of the documentation fails

---


### getSingleModeFlag

```
public abstract boolean getSingleModeFlag()
                                   throws java.lang.Exception
```

:   Return if single mode is set for the given data generator
    mode depends on option setting and or generator type.

    :   **Returns:**: single mode flag **Throws:**: `java.lang.Exception` - if mode is not set yet

---


### setDebug

```
public void setDebug(boolean debug)
```

:   Sets the debug flag.

    :   **Parameters:**: `debug` - the new debug flag

---


### getDebug

```
public boolean getDebug()
```

:   Gets the debug flag.

    :   **Returns:**: the debug flag

---


### debugTipText

```
public java.lang.String debugTipText()
```

:   Returns the tip text for this property

    :   **Returns:**: tip text for this property suitable for displaying in the explorer/experimenter gui

---


### setRelationName

```
public void setRelationName(java.lang.String relationName)
```

:   Sets the relation name the dataset should have.

    :   **Parameters:**: `relationName` - the new relation name

---


### getRelationName

```
public java.lang.String getRelationName()
```

:   Gets the relation name the dataset should have.

    :   **Returns:**: the relation name the dataset should have

---


### relationNameTipText

```
public java.lang.String relationNameTipText()
```

:   Returns the tip text for this property

    :   **Returns:**: tip text for this property suitable for displaying in the explorer/experimenter gui

---


### getNumExamplesAct

```
public int getNumExamplesAct()
```

:   Gets the number of examples the dataset should have.

    :   **Returns:**: the number of examples the dataset should have

---


### setOutput

```
public void setOutput(java.io.PrintWriter newOutput)
```

:   Sets the print writer.

    :   **Parameters:**: `newOutput` - the new print writer

---


### getOutput

```
public java.io.PrintWriter getOutput()
```

:   Gets the print writer.

    :   **Returns:**: print writer object

---


### defaultOutput

```
public java.io.StringWriter defaultOutput()
```

:   Gets the string writer, which is used for outputting to stdout.
    A workaround for the problem of closing stdout when closing the
    associated Printwriter.

    :   **Returns:**: print string writer object

---


### outputTipText

```
public java.lang.String outputTipText()
```

:   Returns the tip text for this property

    :   **Returns:**: tip text for this property suitable for displaying in the explorer/experimenter gui

---


### setDatasetFormat

```
public void setDatasetFormat(Instances newFormat)
```

:   Sets the format of the dataset that is to be generated.

    :   **Parameters:**: `newFormat` - the new dataset format of the dataset

---


### getDatasetFormat

```
public Instances getDatasetFormat()
```

:   Gets the format of the dataset that is to be generated.

    :   **Returns:**: the dataset format of the dataset

---


### formatTipText

```
public java.lang.String formatTipText()
```

:   Returns the tip text for this property

    :   **Returns:**: tip text for this property suitable for displaying in the explorer/experimenter gui

---


### getSeed

```
public int getSeed()
```

:   Gets the random number seed.

    :   **Specified by:**: `getSeed` in interface `Randomizable`
    :   **Returns:**: the random number seed.

---


### setSeed

```
public void setSeed(int newSeed)
```

:   Sets the random number seed.

    :   **Specified by:**: `setSeed` in interface `Randomizable`
    :   **Parameters:**: `newSeed` - the new random number seed.

---


### seedTipText

```
public java.lang.String seedTipText()
```

:   Returns the tip text for this property

    :   **Returns:**: tip text for this property suitable for displaying in the explorer/experimenter gui

---


### getRandom

```
public java.util.Random getRandom()
```

:   Gets the random generator.

    :   **Returns:**: the random generator

---


### setRandom

```
public void setRandom(java.util.Random newRandom)
```

:   Sets the random generator.

    :   **Parameters:**: `newRandom` - is the random generator.

---


### randomTipText

```
public java.lang.String randomTipText()
```

:   Returns the tip text for this property

    :   **Returns:**: tip text for this property suitable for displaying in the explorer/experimenter gui

---


### makeData

```
public static void makeData(DataGenerator generator,
                            java.lang.String[] options)
                     throws java.lang.Exception
```

:   Calls the data generator.

    :   **Parameters:**: `generator` - one of the data generators: `options` - options of the data generator **Throws:**: `java.lang.Exception` - if there was an error in the option list


---


|  |  |  |
| --- | --- | --- |
| **Overview** \| **Package** \| **Class** \| **Tree** \| **Deprecated** \| **Index** \| **Help** \| **Weka's home** | |  |
| **PREV CLASS**   **NEXT CLASS** | **FRAMES**    **NO FRAMES**     **All Classes** |  |
| SUMMARY: NESTED \| FIELD \| CONSTR \| METHOD | DETAIL: FIELD \| CONSTR \| METHOD |  |


---
